# Embryonic stem cell-derived cardiomyocytes for the treatment of doxorubicin-induced cardiomyopathy

**DOI:** 10.1186/s13287-018-0788-2

**Published:** 2018-02-05

**Authors:** Danúbia Silva dos Santos, Guilherme Visconde Brasil, Isalira Peroba Rezende Ramos, Fernanda Cristina Paccola Mesquita, Tais Hanae Kasai-Brunswick, Michelle Lopes Araújo Christie, Gustavo Monnerat Cahli, Raiana Andrade Quintanilha Barbosa, Sandro Torrentes da Cunha, Jonathas Xavier Pereira, Emiliano Medei, Antonio Carlos Campos de Carvalho, Adriana Bastos Carvalho, Regina Coeli dos Santos Goldenberg

**Affiliations:** 1 Instituto de Biofísica Carlos Chagas Filho, Universidade Federal do Rio de Janeiro, Av. Carlos Chagas Filho 373 Bloco G—Sala G2-053, Rio de Janeiro, RJ 21941-902 Brazil; 2 Centro Nacional de Biologia Estrutural e Bioimagem, Universidade Federal do Rio de Janeiro, Av. Carlos Chagas Filho, 373, Bloco M, Rio de Janeiro, RJ 21941-902 Brazil; 3 Departamento de Patologia—Faculdade de Medicina, Hospital Universitário Clementino Fraga Filho, Universiade Federal do Rio de Janeiro, Av. Rodolpho Paulo Rocco, 255, Sub-solo, SAP, Rio de Janeiro, RJ 21910-590 Brazil; 4 Instituto Nacional de Ciência e Tecnologia em Medicina Regenerativa, Av. Carlos Chagas Filho 373, Rio de Janeiro, RJ 21941-902 Brazil

**Keywords:** Dox-induced cardiomyopathy, Heart failure, Cardiomyocytes derived from mouse embryonic stem cells, Cell therapy

## Abstract

**Background:**

Doxorubicin (Dox) is a chemotherapy drug with limited application due to cardiotoxicity that may progress to heart failure. This study aims to evaluate the role of cardiomyocytes derived from mouse embryonic stem cells (CM-mESCs) in the treatment of Dox-induced cardiomyopathy (DIC) in mice.

**Methods:**

The mouse embryonic stem cell (mESC) line E14TG2A was characterized by karyotype analysis, gene expression using RT-PCR and immunofluorescence. Cells were transduced with luciferase 2 and submitted to cardiac differentiation. Total conditioned medium (TCM) from the CM-mESCs was collected for proteomic analysis. To establish DIC in CD1 mice, Dox (7.5 mg/kg) was administered once a week for 3 weeks, resulting in a cumulative Dox dose of 22.5 mg/kg. At the fourth week, a group of animals was injected intramyocardially with CM-mESCs (8 × 10^5^ cells). Cells were tracked by a bioluminescence assay, and the body weight, echocardiogram, electrocardiogram and number of apoptotic cardiomyocytes were evaluated.

**Results:**

mESCs exhibited a normal karyotype and expressed pluripotent markers. Proteomic analysis of TCM showed proteins related to the negative regulation of cell death. CM-mESCs presented ventricular action potential characteristics. Mice that received Dox developed heart failure and showed significant differences in body weight, ejection fraction (EF), end-systolic volume (ESV), stroke volume (SV), heart rate and QT and corrected QT (QTc) intervals when compared to the control group. After cell or placebo injection, the Dox + CM-mESC group showed significant increases in EF and SV when compared to the Dox + placebo group. Reduction in ESV and QT and QTc intervals in Dox + CM-mESC-treated mice was observed at 5 or 30 days after cell treatment. Cells were detected up to 11 days after injection. The Dox + CM-mESC group showed a significant reduction in the percentage of apoptotic cardiomyocytes in the hearts of mice when compared to the Dox + placebo group.

**Conclusions:**

CM-mESC transplantation improves cardiac function in mice with DIC.

**Electronic supplementary material:**

The online version of this article (10.1186/s13287-018-0788-2) contains supplementary material, which is available to authorized users.

## Background

Anthracyclines are among the most effective anticancer agents ever developed, with proven effects on various solid and hematological tumors [1, 2]. Doxorubicin (Dox), the most frequently prescribed anthracycline, has been used mainly for the treatment of breast cancer, solid tumors in children, soft tissue sarcomas and aggressive lymphomas [2]. However, the use of anthracyclines may have severe toxic effects, such as anthracycline-induced cardiomyopathy.

The toxicity of anthracyclines may vary in different tissues and depends on the duration of exposure. Some of the observed side effects (nausea, vomiting, myelosuppression, mucositis, among others) are expected and considered a byproduct of the drug mechanism of action, and may disappear spontaneously or with changes in the scheduling of chemotherapy cycles or after treatment is completed [3]. However, the cardiotoxic effects of anthracyclines generate changes in myocardial structure, which can develop into severe and irreversible cardiomyopathy [3, 4]. Anthracycline-induced cardiomyopathy is known for impacting the quality of life of patients, especially children, who have survived cancer [3, 4]. According to Raj et al. [4], patients diagnosed with cancer during childhood who are treated with Dox are at high risk of cardiac events up to 30 years post treatment. Furthermore, it is estimated that one in eight patients treated with anthracyclines will have severe heart disease [4].

Multiple molecular and pathophysiological mechanisms have been proposed to explain anthracycline-induced cardiotoxicity, including increased oxidative stress, inhibition of nucleic acid and protein synthesis, structural and functional changes of myofibrils and changes in iron metabolism, among others [2, 5–7].

Dox is known to reduce the expression of cardiac genes, including GATA4, α-sarcomeric actin, myosin light and heavy chains, troponin I and C, titin, Ca^+2^-ATPase and ryanodine receptor, due to changes in transcriptional regulatory proteins [2, 7]. Some evidence suggests that anthracyclines induce apoptosis of cardiomyocytes and endothelial cells. Thus, the loss of cardiomyocytes may initiate and/or exacerbate heart failure [2, 5, 6].

Cardiotoxicity may be aggravated by the cumulative dose of anthracyclines and by fast drug infusion [3, 4, 8–11]. It is well known that the damage caused by the cumulative dose of anthracyclines is associated with repeated administration of the drug, which can cause early diastolic dysfunction and late systolic dysfunction, limiting the extent to which these drugs can be used safely [5].

Despite our improved understanding of the alterations that occur in the heart, there is still no treatment for the injuries caused by anthracyclines.

Considering that anthracyclines may induce cardiomyocyte death through the activation of different molecular and pathophysiological mechanisms, this study investigated whether cardiomyocytes derived from mouse embryonic stem cells (CM-mESCs) can be integrated into the damaged heart tissue and recover cardiac function in a model of Dox-induced cardiomyopathy (DIC) in CD1 mice.

## Methods

### mESC culture

Mouse embryonic stem cell (mESC) line E14TG2A was kindly donated by Dr Henrique Marques Souza (University of Campinas, Campinas, SP, Brazil). Cells were plated on gelatinized tissue culture plates in Glasgow Minimum Essential Medium (GMEM; Sigma-Aldrich) supplemented with 15% (v/v) fetal bovine serum (FBS; Gibco), 2 mM l-glutamine (Sigma-Aldrich), 50 U/ml penicillin–streptomycin (Gibco), 1% (v/v) nonessential amino acids (Gibco), 0.1 mM β-mercaptoethanol (Gibco), 1% (v/v) sodium pyruvate and 10^3^ U/ml leukemia inhibitory factor (LIF; Gibco) at 37 °C under 5% CO_2_ and 95% humidity. The cells were passaged every 3 days by enzymatic dissociation with 0.25% trypsin–EDTA (Gibco). When passages were carried out, cells were transferred from one to two plates (100 mm; Corning). Cultured medium was changed daily.

### Karyotype analysis

For detection of aneuploidy, a chromosome preparation was performed. E14TG2A-mESCs were arrested in metaphase with 0.1 μg/ml KaryoMAX^®^ Colcemid™ solution (Life Technologies) for 90 minutes, dissociated from culture flasks with 0.25% trypsin–EDTA (Gibco) and incubated in hypotonic solution with 57 mM KCl (Merck) for 20 minutes at 37 °C. Cells were collected after centrifugation (170 × *g* for 8 minutes) and fixed with a methanol–acetic acid solution (3:1; Merck). Chromosome spreads were obtained by pipetting suspension drops onto clean glass slides. Metaphase cells were stained using Wright’s eosin methylene blue (Merck), and 20 metaphases were karyotyped for each sample (*n* = 3).

### Reverse transcription-polymerase chain reaction

Total RNA was extracted from the cells using an RNeasy Mini Kit (Qiagen) following the manufacturer’s instructions. One microgram of total RNA was reverse transcribed into cDNA using random primers and a High-Capacity Reverse Transcription Kit (Applied Biosystems) following the manufacturer’s instructions. The sequences of primers and sizes of expected products are presented in Table 1. Aliquots (500 ng) of each cDNA sample were amplified in a Peltier Thermal Cycler PTC-200 (MJ Research) in a 20-μl reaction mixture containing 1 × PCR Buffer (Promega), 2.5 mM MgCl_2_, 0.2 mM each of deoxynucleotide triphosphates (dNTPs), 0.2 mM each of sense and antisense primers, and 1.25 units of Go TaqR DNA Polymerase (Promega). The PCR program consisted of denaturation at 95 °C for 5 minutes, 30 cycles of denaturation at 95 °C for 1 minute, annealing at 56 °C for 1 minute and extension at 72 °C for 1 minute, followed by a final extension at 72 °C for 10 minutes. The PCR products were analyzed on a 2% agarose gel (Sigma-Aldrich) and revealed using ethidium bromide (Sigma-Aldrich).

**Table 1:** Primers used for reverse transcription-polymerase chain reaction to establish the undifferentiated state of mouse embryonic stem cell line E14TG2A

Gene product	Sense primer sequence (orientation: 5′–3′)	Antisense primer sequence (orientation: 3′–5′)	Size (base pairs)
*Oct3/4*	AGCCTGAGGGCGAAGCAGGA	CCCCAGGGTGAGCCCCACAT	236
*Sox2*	AGCTACAGCATGATGCAGGA	GGTCATGGAGTTGTACTGCA	126
*Nanog*	CAGCCCTGATTCTTCCACCAGTCCC	TGGAAGGTTCCCAGTCGGGTTCACC	391
*β-actin*	CATCACTATTGGCAACGAGCG	ATGGATGCCACAGGATTCCA	85

### Immunofluorescence

The mESCs were fixed in 4% (v/v) paraformaldehyde for 20 minutes at room temperature and permeabilized with 0.3% (v/v) Triton X-100. First, fixed cells were incubated for 30 minutes at room temperature with 2% bovine serum albumin (BSA; Sigma) to reduce nonspecific binding. Then, cells were incubated overnight at 4 °C with primary antibodies against octamer-binding transcription factor 3/4 (OCT3/4) (ab-19857, diluted 1:500; Abcam) and stage-specific embryonic antigen-1 (SSEA-1 (480)) (sc-21702; diluted 1:100; Santa Cruz Biotechnology). Next, cells were incubated for 1 hour at room temperature with Cy3-AffiniPure donkey anti-rabbit (711-165-152, diluted 1:800; Jackson Immunoresearch) and Alexa Fluor^®^ 488-conjugated goat anti-mouse (A21042, diluted 1:400; Life Technologies) secondary antibodies. The coverslips were then washed three times for 10 minutes each with PBS, the nuclei were stained with Topro^®^-3 (diluted 1:1000; Life Technologies) and coverslips were mounted in antifading solution VECTASHIELD H-1000 (Vector Laboratories). Fluorescence was observed using a confocal microscope (Zeiss LSM 510 Meta) coupled to a system for capturing digital photomicrographs. The specificity of the immunofluorescent staining was assessed for each experimental condition by performing the reaction in the absence of primary antibodies.

### mESC transduction with luciferase 2

Lentiviral vector pMSCV.Luc2.T2A.Puro was constructed as described previously [12]. Lentiviral particles containing luciferase 2 (Luc2) and puromycin resistance genes were produced in HEK 293FT cells with the pMSCV.Luc2.T2A.Puro vector and the accessory vectors pΔ8.9 and pHDM-VSV-G, using the transfection reagent FuGene 6 (Roche). At 24, 36 and 48 hours after transfection, culture media containing lentiviral particles were filtered (0.22 μm; Merck Millipore^®^) and centrifuged at 20,000 × *g*. mESCs were cultivated with polybrene (8 μg/ml; Merck Millipore^®^) and lentiviral particles. After 24 hours of incubation, culture medium was replaced. Approximately 60 hours after transduction, 1.0 μg/ml puromycin was added to the medium. Cells were selected for 4 days and then expanded for bioluminescence imaging assay.

### Cardiac differentiation

mESCs were submitted to cardiac differentiation according to Chen *et al.* [13]. mESCs were dissociated by 0.25% trypsin–EDTA (Gibco) and cultured using the hanging drop (HD) method to form embryoid bodies (EBs). Approximately 600 cells in each 20-μl drop of differentiation medium (high glucose (4.5 g/l) Dulbecco’s Modified Eagle’s medium (DMEM; Gibco) supplemented with 20% (v/v) FBS, 2 mM l-glutamine (Sigma-Aldrich), 50 U/ml penicillin–streptomycin (Gibco), 1% (v/v) nonessential amino acids (Gibco), 0.1 mM β-mercaptoethanol (Gibco), 2 μM dorsomorphin dihydrochloride (Tocris Bioscience) and 1% dimethyl sulfoxide (DMSO; Sigma-Aldrich)) were plated on the lids of 100-mm plates (Corning) and cultured using the HD technique for 2 days. Subsequently, EBs were cultured in suspension in 60-mm plates (Corning) coated with poly 2-hydroxyethyl methacrylate (Sigma-Aldrich) in the same differentiation medium described earlier, excluding dorsomorphin, for 3 days. EBs were transferred to 0.1% (v/v) gelatin-coated dishes (35 mm; Corning) and cultured in differentiation medium, without dorsomorphin and DMSO, for another 10 days.

### Flow cytometry

On the 14th day, differentiated cells were dissociated with 0.25% trypsin–EDTA. For intracellular staining, cells were fixed in 4% paraformaldehyde for 20 minutes at room temperature and permeabilized with 0.3% Triton X-100 in PBS for 30 minutes. Cells were blocked with 0.5% (v/v) BSA in PBS and stained with troponin T cardiac isoform Ab-1 (MS-295P1, diluted 1:200; Thermo Scientific™) for 30 minutes at room temperature. Subsequently, cells were incubated for 30 minutes at room temperature with Alexa Fluor^®^ 647-conjugated goat anti-mouse (A21236, diluted 1:1000; Life Technologies). Permeabilized cells were selected by DAPI staining and samples were analyzed using BD FACSAria IIu and FlowJo v10 software.

### Preparation of conditioned media

On day 15 of the differentiation protocol, CM-mESCs at 85–90% confluence (~ 1 × 10^6^ cells) were washed with PBS and incubated in differentiation medium without FBS. After 24 hours, the medium was collected, which represents the total conditioned medium (TCM). The TCM was cleared of cellular debris and particulate matter by centrifugation at 1000 × *g* for 30 minutes followed by 20,000 × *g* for 30 minutes, and then concentrated using 3-kDa filters (Merck Millipore^®^) as described previously [14].

### Protein identification using liquid chromatography tandem mass spectrometry

#### Sample preparation

TCM was prepared as already described. All steps were performed on ice and with protease inhibitor cocktail (Roche) to avoid degradation of proteins. Briefly, protein samples were precipitated with 10% (1:4 v/v) trichloroacetic acid (Sigma-Aldrich) in acetone (Sigma-Aldrich) by incubation on ice overnight followed by centrifugation for 15 minutes at 4 °C at 15,000 rpm. Samples were washed three times with cold acetone and air dried. Proteins were suspended in 15 μl of 7 M urea/2 M thiourea (Sigma-Aldrich) and were quantified using a Quibit Protein Assay Kit (Thermo Scientific™). Proteins were reduced with 10 mM dithiothreitol (DTT; Sigma-Aldrich) by incubation for 1 hour at 30 °C followed by alkylation by incubation with 55 mM iodoacetamide (IAM; Sigma-Aldrich) for 30 minutes in the dark at room temperature. After alkylation, 50 mM NH_4_HCO_3_ (10:1 v/v; Sigma-Aldrich) and mass spectrometry-grade trypsin (Promega) at a ratio of 50:1 (protein:trypsin) were added to the samples, and samples were incubated overnight at 35 °C. After digestion, the samples were acidified using 0.1% trifluoroacetic acid (TFA; Sigma-Aldrich). Samples were cleaned with an in-house prepared C-18 column and eluted in 50 μl of 50% acetonitrile (ACN)/0.1% TFA followed by 50 μl of 70% ACN/0.1% TFA, dried in a SpeedVac concentrator (Thermo Scientific™) and resuspended in 15 μl of 0.1% formic acid (Sigma-Aldrich). Peptides were quantified using a Quibit Protein Assay Kit and suspended to a final concentration of 0.25 μg/μl in 0.1% formic acid.

#### Sample analysis and protein identification

Samples were analyzed in three technical replicates by liquid chromatography tandem mass spectrometry (LC-MS/MS) as described previously [15]. Briefly, 4 μl of the diluted samples were applied to an EASY II nano LC system (Proxeon Biosystem) coupled online to an ESI-LTQ Orbitrap Velos mass spectrometer (Thermo Scientific™). Peptides were loaded into a trap column (150 μm × 2 cm) packed in-house with C-18 ReproSil 3-μm resin (Dr. Maisch, GmbH, Germany) and eluted in an analytical column (100 μm × 15 cm) packed with the same material. Peptide separations were performed using a gradient from 100% solution A (0.1% formic acid) to 35% solution B (0.1% formic acid, 95% acetonitrile; Sigma-Aldrich) over 100 minutes followed by 35–90% solution B over 15 minutes, and were maintained in 90% solution B for 5 minutes. MS1 spectra were acquired in a positive mode using the data-dependent automatic (DDA) survey MS scan. Each DDA consisted of a survey scan in the *m*/*z* range of 300−2000 and a resolution of 60,000 with a target value of 1 × 10^–6^ ions. The 14 most intense ions were subjected to MS2 acquisition in the LTQ using normalized collision-induced dissociation (CID) of previously fragmented ions.

MS data were analyzed with PatternLab (version 4.0.0.62) using a Uniprot *Mus Musculus* database downloaded in June 2017. Search parameters were semi-tryptic hydrolysis, two missed cleavages, oxidation of methionine as variable and carbamidomethylation as fixed modifications, and a peptide tolerance of 40 ppm. The validity of the peptide sequence matches (PSMs) was assessed using Search Engine Processor version 2.2.0.2 (SEPro) software. A cutoff score was established to accept a false discovery rate (FDR) of 1% based on the number of decoys. A minimum sequence length of six amino acids was required. The results were postprocessed to accept PSMs with less than 5 ppm. Proteins were identified according to the maximum parsimony approach. The identified proteins were submitted to the Gene Ontology Biological Process in the STRING online database [16], and biological processes related to cardiovascular recovery were selected.

### Electrophysiology and action potential recordings

The 35-mm plates containing CM-mESCs were transferred to the stage of an inverted microscope (Nikon). Action potentials were recorded as described previously [17]. CM-mESC preparations were superfused with Tyrode’s solution containing 150.8 mM NaCl, 5.4 mM KCl, 1.8 mM CaCl_2_, 1.0 mM MgCl_2_, 11.0 mM d-glucose and 10.0 mM HEPES (pH 7.4 adjusted with NaOH). The superfusion was carried out at 37.0 ± 0.5 °C using a temperature controller (Harvard Apparatus) saturated with oxygen at a perfusion flow rate of 0.5 ml/minute (Miniplus 3). Transmembrane potential was recorded using glass microelectrodes (40–80 MΩ DC resistance) filled with 2.7 M KCl connected to a microelectrode amplifier (MultiClamp 700B; Molecular Devices, USA). Amplified signals were digitized (1440 Digidata A/D interface) and stored on a computer for future analysis using LabChart 7.3 software (ADInstruments). The following parameters were analyzed: resting membrane potential (RMP), action potential amplitude (APA) and action potential duration at 30, 50, 70 and 90% repolarization (APD30, APD50, APD70 and APD90, respectively) for 10 consecutive action potentials from each cell (*n* = 8).

### Animals

Experiments were performed in 16-week-old female and male immunocompetent CD1 mice weighing 25–42 g. All experiments were performed in conformity with the *Guide for the Care and Use of Laboratory Animals* (National Institutes of Health) and were approved by the Ethics Committee for Animal Use of the Federal University of Rio de Janeiro under number IBCCF 171/13. Mice were housed in a controlled temperature (23 °C) environment with a 12:12-hour light/dark cycle and received standard mouse chow and water *ad libitum*.

### Establishment of Dox-induced cardiomyopathy and cell transplantation

**Fig. 1:**
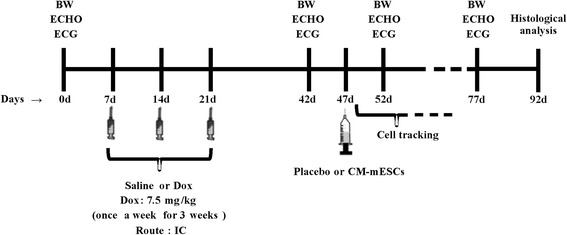
Experimental design. Schematic timeline of the study design. BW body weight, ECHO echocardiogram, ECG electrocardiogram, Dox doxorubicin, IC intracavitary, CM-mESC cardiomyocytes derived from mouse embryonic stem cell

Dox (7.5 mg/kg) was injected in mice once a week for 3 weeks using 29-G needles (BD Ultra-Fine) via an intracavitary route (IC; left ventricle cavity) guided by echocardiogram (ECHO) for a cumulative dose of 22.5 mg/kg. Control mice received an equivalent volume of saline. Before administration of Dox or saline, animals were anesthetized with 1.5% isoflurane (Cristalia^®^) in O_2_. Doxorubicin hydrochloride (Adriblastin^®^ RD) was dissolved in sterile 0.9% saline solution. CD1 mice were divided into two groups: the Dox group (*n* = 32) and the control group (saline solution; *n* = 18). Body weight and cardiac mechanical and electrical functions were evaluated at day 0 (before Dox and saline injection), day 42 (3 weeks after the last injection), day 52 and day 77 (5 and 30 days after cell or placebo injection, respectively; Fig. 1).

Out of the 32 animals treated with Dox, two mice died during the drug injection procedure and three did not develop DIC (determined by an ejection fraction (EF) < 50% at day 42). The 27 remaining animals were distributed into two groups, the Dox + CM-mESC group (*n* = 14) and the Dox + placebo group (*n* = 13), which were treated via ECHO-guided intramyocardial injections of 30 μl of PBS containing either 8 × 10^5^ CM-mESCs or just PBS at day 47 (4 weeks after chemotherapy). After treatment, four animals from the Dox + CM-mESC group died during the anesthetic procedure for the bioluminescence imaging assay. The Dox + placebo animals were not submitted to this procedure.

### Assessment of cardiac mechanical and electrical function

The cardiac function parameters of EF, end-diastolic volume (EDV) and end-systolic volume (ESV) corrected by body weight (BW), and stroke volume (SV) were determined by ECHO imaging using Simpson’s method. For ECHO examinations, mice were anesthetized with 1.5% isoflurane in O_2_ and the thoracic region was shaved. A small gel standoff pad was placed between the chest and a 30-MHz ultrasound scanhead connected to a Vevo 770 Imaging System (VisualSonics). Heart rate (HR) and body temperature were monitored during examinations.

The electrical activity parameters of HR, PR, QT and corrected QT (QTc) intervals, and P-wave and QRS durations were determined using an electrocardiograph (PowerLab/400, speed 25 mm/s; ADInstruments). For electrocardiogram (ECG) examinations, mice were gently placed on the ECG recording platform equipped with three electrodes configured to contact the underside of their paws. ECG signals were collected for 2 minutes per mouse. The ECG recording was analyzed using LabChart 7.3 software (ADInstruments).

### Bioluminescence imaging assay

Bioluminescence imaging assay was performed as described previously [18]. For *in-vitro* studies, transduced cells were plated in a 24-well plate at different concentrations: 4 × 104, 6 × 104, 8 × 104, 10 × 104, and 12 × 10^4^ CM-mESCs per well. d-Luciferin (150 μg/ml; Promega Corporation) was added to the culture medium. The culture plate was immediately positioned in the IVIS Lumina Imaging System (Caliper Life Sciences) and images were acquired after a 10-second exposure period.

For *in-vivo* studies, mice received d-Luciferin (150 mg/kg) intraperitoneally. Ten minutes after injection, they were anesthetized with 1.5% isoflurane in O_2_ and placed in the IVIS Lumina Imaging System. Image acquisitions were performed at 1, 2, 4, 6, 8, and 11 days after the injection of transduced CM-mESCs. The exposure time was 3 minutes.

For *ex-vivo* studies, mice received d-Luciferin (150 mg/kg) intraperitoneally. Animals were anesthetized with 1.5% isoflurane in O_2_ and euthanized on days 1, 4 and 11, 10 minutes after d-Luciferin injection. The organs were removed and placed in a 24-well plate with PBS for imaging.

Results were analyzed using Living Image Software 3.2 (Caliper Life Sciences).

### Cell tracking by luciferase 2 expression

To confirm the engraftment of transduced CM-mESCs in the heart, total DNA was extracted from the left ventricle of mice in the Dox + CM-mESC group at 1, 4, 11 and 45 days after cell injection. DNA from 25 mg of cardiac tissue was isolated using a DNeasy Blood & Tissue kit (Qiagen) according to the manufacturer’s instructions. DNA yield and quality were accessed using a NanoDrop 2000 spectrophotometer (Thermo Scientific™). The presence of the luciferase 2 gene in samples was identified by polymerase chain reaction (PCR) using specific primers (forward, 5′-ACCAGGGCTTCCAAAGCATGTAC-3′; reverse 5′-CGGGCATGACTGAATCGGACAC-3′). The thermal cycler conditions were an initial stage at 94 °C for 2 minutes, followed by 35 cycles of denaturation at 94 °C for 30 seconds, annealing at 60 °C for 30 seconds and extension at 72 °C for 30 seconds, and a final stage at 72 °C for 10 minutes. Transduced mESCs were used as positive control. The PCR products were subjected to 2% agarose gel electrophoresis, revealed using ethidium bromide and photographed using an imaging system (Odyssey^®^ Fc; LI-COR Biosciences).

### Histology

At 45 days after cell injection, mice were euthanized and hearts were perfused with 4% paraformaldehyde. Cardiac tissue was embedded in paraffin and 8-μm slices were obtained. Slices were stained with Sirius Red. Fibrosis was quantified in 30 random fields as a percentage of the total area of cardiac tissue using Image-Pro Plus 5.0 software.

### Identification of apoptotic cardiomyocytes

Apoptotic cells were evaluated by terminal deoxynucleotidyl transferase-mediated 2′-deoxyuridine 5′-triphosphate nick end labeling (TUNEL) assay with an *in-situ* cell death detection kit (ApopTag^®^ Peroxidase *In Situ* Apoptosis Detection Kit S7100; Merck Millipore^®^). Positive nuclei were visualized with 3,3′-diaminobenzidine (DAB; Spring Bioscience) staining. Next, we identified TUNEL-positive apoptotic cardiomyocyte nuclei. In brief, TUNEL stained sections were stained with cardiac troponin T antibody (ab80050, diluted 1:100; Abcam). Following incubation, sections were washed with PBS and labeled with goat anti-rabbit IgG-AP (sc-2034, diluted 1:200; Santa Cruz) secondary antibody. Negative nuclei were stained with 1.0% (v/v) Safranin O (s2255-25 g; Sigma-Aldrich). Sections were mounted with Entellan^®^ New (Merck Millipore^®^) and examined with a phase-contrast optical microscope (Zeiss Axioplan). Brown nuclei (apoptotic), red nuclei (nonapoptotic) and blue cardiomyocytes were automatically quantified in five random areas in one or two heart sections from three different hearts using TMARKER software.

### Statistical analysis

All values are expressed as the mean ± standard deviation. Cardiac function parameters and BW were analyzed using two-way analyses of variance (ANOVAs) followed by Bonferroni multiple comparisons tests. For histological and apoptosis analyses, the percentage of Sirius Red-stained area, apoptotic cells and apoptotic cardiomyocytes were analyzed using one-way ANOVA with Bonferroni multiple comparisons test. Differences between variables were considered significant when *P* < 0.05. GraphPad Prism^®^ software version 5.0 (GraphPad Software, Inc., La Jolla, CA, USA) was used for all statistical analyses.

## Results

### Cell culture and differentiation

**Fig. 2:**
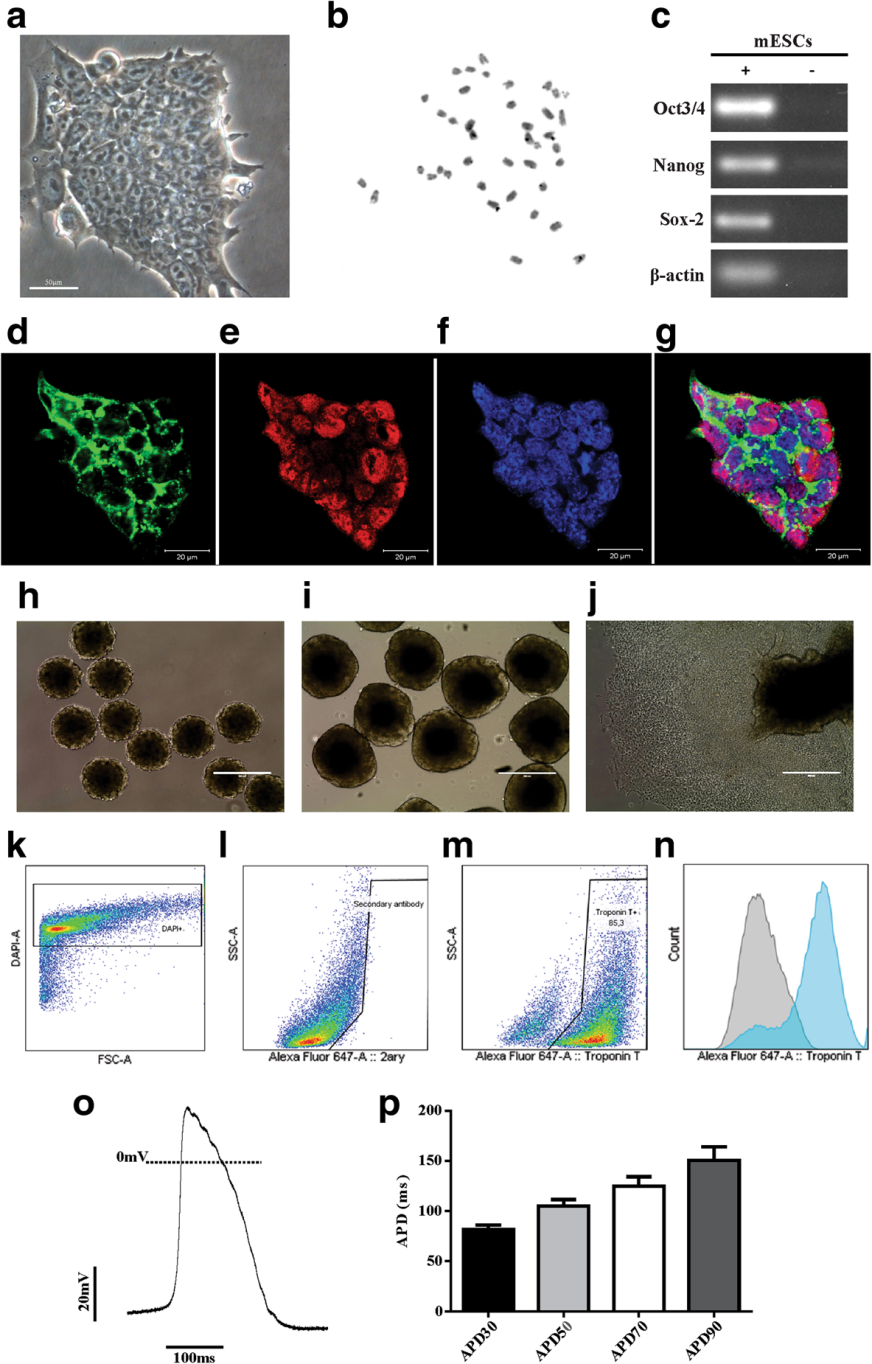
Characterization of undifferentiated mESC line E14TG2A, embryoid body formation and cardiac differentiation. **a** mESCs in culture. **b** Karyotype analysis showing 40 chromosomes. **c** Expression of undifferentiated mESC transcription factors by RT-PCR: Oct3/4, Nanog and Sox-2. **d**–**g** Expression of pluripotency markers in mESCs by immunofluorescence: cells positive for **d** SSEA-1 (green) and **e** Oct3/4 (red); **f** nuclei labeled with Topro (blue); and **g** merged image. **h, i** Embryoid bodies (EBs) in suspension after 2 and 5 days of differentiation. **j** Adhered EBs after 8 days of differentiation with beating cells. **k**–**n** Flow cytometry analysis on day 14: representative graphs of **k** DAPI-positive differentiated cells used to isolate permeabilized cells only, **l** differentiated cells stained only with the secondary antibody and **m** cardiac troponin T-positive differentiated cells; and **n** histogram overlay of differentiated cells stained with secondary antibody (gray) and cells expressing cardiac troponin t (blue). **o** Representative trace of ventricular action potential. **p** Action potential duration at 30, 50, 70 and 90% of repolarization (APD30, APD50, APD70 and APD90, respectively; *n* = 8). Scale bars: **a** 50 μm, **d**–**g** 20 μm. mESC mouse embryonic stem cell, *DAPI* 4′,6-diamidino-2-phenylindole, FSC forward scatter, SSC side scatter

E14TG2A mESC colonies showed morphological characteristics of undifferentiated embryonic stem cells, with bright edges, high nuclear-cytoplasmic ratio and little spacing between cells (Fig. 2a). We did not observe any karyotypic alterations (Fig. 2b). Amplification of OCT3/4, Nanog and Sox-2 was successful (Fig. 2c), and the expression of OCT3/4 and SSEA-1 in mESCs was analyzed (Fig. 2d–g).

Next, we initiated the cardiac differentiation protocol. After 2 days, mESCs formed EBs of similar size that were homogeneous with well-defined borders (Fig. 2h). After 5 days, we observed larger EBs in suspension (Fig. 2i). On the 8th day, we observed adhered cells migrating from the central region of EBs to the periphery (Fig. 2j). We also detected that the first differentiated cells showed spontaneous contraction. In addition, we examined the efficiency of the differentiation protocol. After 14 days, differentiated cells presented high percentages of cTnT-positive cells (Fig. 2k–n). Five independent experiments of cardiac differentiation were conducted, and an average of 80.6% cells were positive for cTnT (Table 2). We observed that CM-mESCs showed ventricular action potential morphology (Fig. 2o). CM-mESCs had APD values similar to those of ventricular cells, with APD30 of 81.1 ms, APD50 of 104.8 ms, APD70 of 124.9 ms and APD90 of 150.5 ms (Fig. 2p).

**Table 2:** Efficiency of the differentiation protocol of mESC E14TG2A-luc2 into cardiomyocytes

Differentiation	Cardiac troponin T-positive cells (%)
1	83.2
2	60.3
3	77.9
4	86.3
5	95.3
Mean	80.6
SD	12.98

Table presents results of five independent differentiation experiments and the percentage of cardiac troponin T-positive cells (80.6 ± 12.98, mean ± SD) as determined by fluorescence-activated cell sorting analysis

*mESC* mouse embryonic stem cell, luc2 luciferase 2, *SD* standard deviation

### Dox-induced cardiomyopathy and therapy with CM-mESCs

**Fig. 3:**
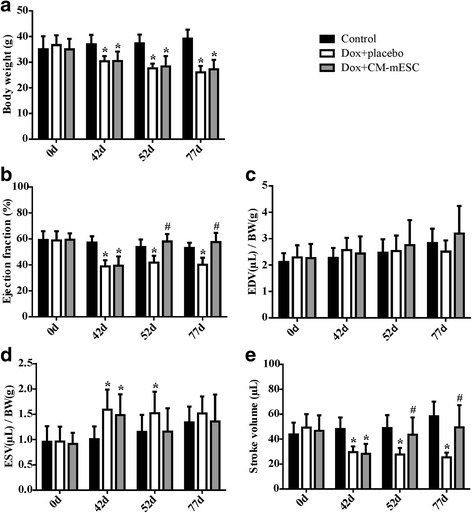
Assessment of cardiac function in DIC mice transplanted with CM-mESCs or placebo. Measures performed before (corresponding to day 0) and after (corresponding to day 42) Dox or saline administration, and after cell or placebo injection (corresponding to days 52 and 77, respectively). **a** Analysis of body weight (BW), **b** ejection fraction (EF), **c, d** end-diastolic volume (EDV) and end-systolic volume (ESV) corrected by BW, and **e** stroke volume (SV). Two-way ANOVA with Bonferroni post test: *p < 0.05 compared to the control group; #*p* < 0.05 compared to the Dox + placebo group. Data shown as mean ± standard deviation; *n* = 18 for the control group, *n* = 13 for the Dox + placebo group and *n* = 10 for the Dox + CM-mESC group. Dox doxorubicin, CM-mESC cardiomyocytes derived from mouse embryonic stem cell, days

**Fig. 4:**
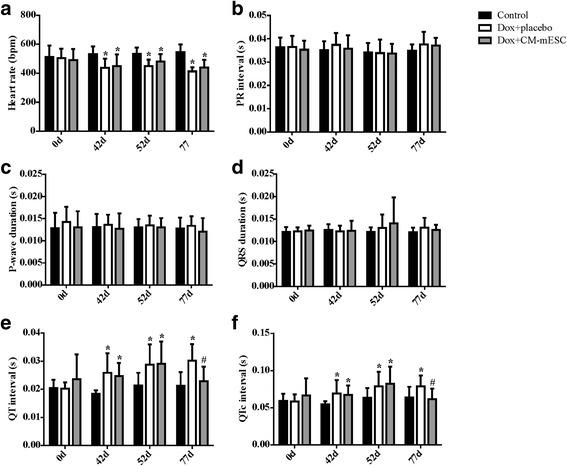
Assessment of electrical activity in DIC mice transplanted with CM-mESCs or placebo. Measures performed before (corresponding to day 0) and after (corresponding to day 42) Dox or saline administration, and after cell or placebo injection (corresponding to days 52 and 77, respectively). **a** Analysis of heart rate (HR), **b** PR interval, **c** P-wave duration, **d** QRS duration, **e** QT interval, and **f** QTc interval. Two-way ANOVA with a Bonferroni post test: *p < 0.05 compared to the control group; #*p* < 0.05 compared to the Dox + placebo group. Data shown as mean ± standard deviation; *n* = 18 for the control group, *n* = 13 for the Dox + placebo group and *n* = 10 for the Dox + CM-mESC group. Dox doxorubicin, CM-mESC cardiomyocytes derived from mouse embryonic stem cell, d days, QTc corrected QT

Since CM-mESCs had a ventricular phenotype and considering that Dox may induce cardiomyocyte death, we considered CM-mESCs a preferable source for cell therapy in our model. On day 42 (corresponding to 21 days after the end of chemotherapy), before cell or placebo treatment, we observed a significant reduction in the BW of the Dox + placebo and the Dox + CM-mESC animals (Fig. 3a). Using ECHO and ECG, we evaluated cardiac function and electrical activity in the hearts of mice with DIC. The analysis showed significant differences in EF, ESV/BW, SV, HR and QT and QTc intervals of both groups when compared to the control group (Fig. 3b, d, e and Fig. 4a, e, f, respectively). No differences were observed in EDV/BW, PR interval or P-wave and QRS duration between groups (Fig. 3c and Fig. 4b–d, respectively). In addition, no significant differences were observed in these parameters between mice in the Dox + placebo group and mice in the Dox + CM-mESC group at this time point.

On days 52 and 77 of the study design (corresponding to 5 and 30 days after placebo or cell therapy, respectively), no difference was observed in the BW between the Dox + placebo group and the Dox + CM-mESC group (Fig. 3a). In addition, we observed that the BWs of mice of both groups remained lower when compared with the control group. We observed significant increases in EF and SV in mice from the Dox + CM-mESC group when compared with those in the Dox-placebo group (Fig. 3b, e). The ESV/BW ratio in the Dox + CM-mESC group on day 52 was similar to that in the control group, but this parameter was significantly increased in the Dox + placebo group when compared to the control group (Fig. 3d). When the QT and QTc intervals were examined, we observed a reduction in these parameters in the Dox + CM-mESC group on day 77 when compared to the Dox-placebo group (Fig. 4e, f). No differences were observed in the QT and QTc intervals between the Dox + CM-mESC group and the control group at this time point.

We observed that EF, ESV/BW, SV, and the QT and QTc intervals were significantly different between the Dox + placebo group and the control group on days 52 and 77, except for ESV/BW on day 77 (Fig. 3b, d, e and Fig. 4e, f, respectively).

### Distribution of CM-mESCs after cell transplantation

**Fig. 5:**
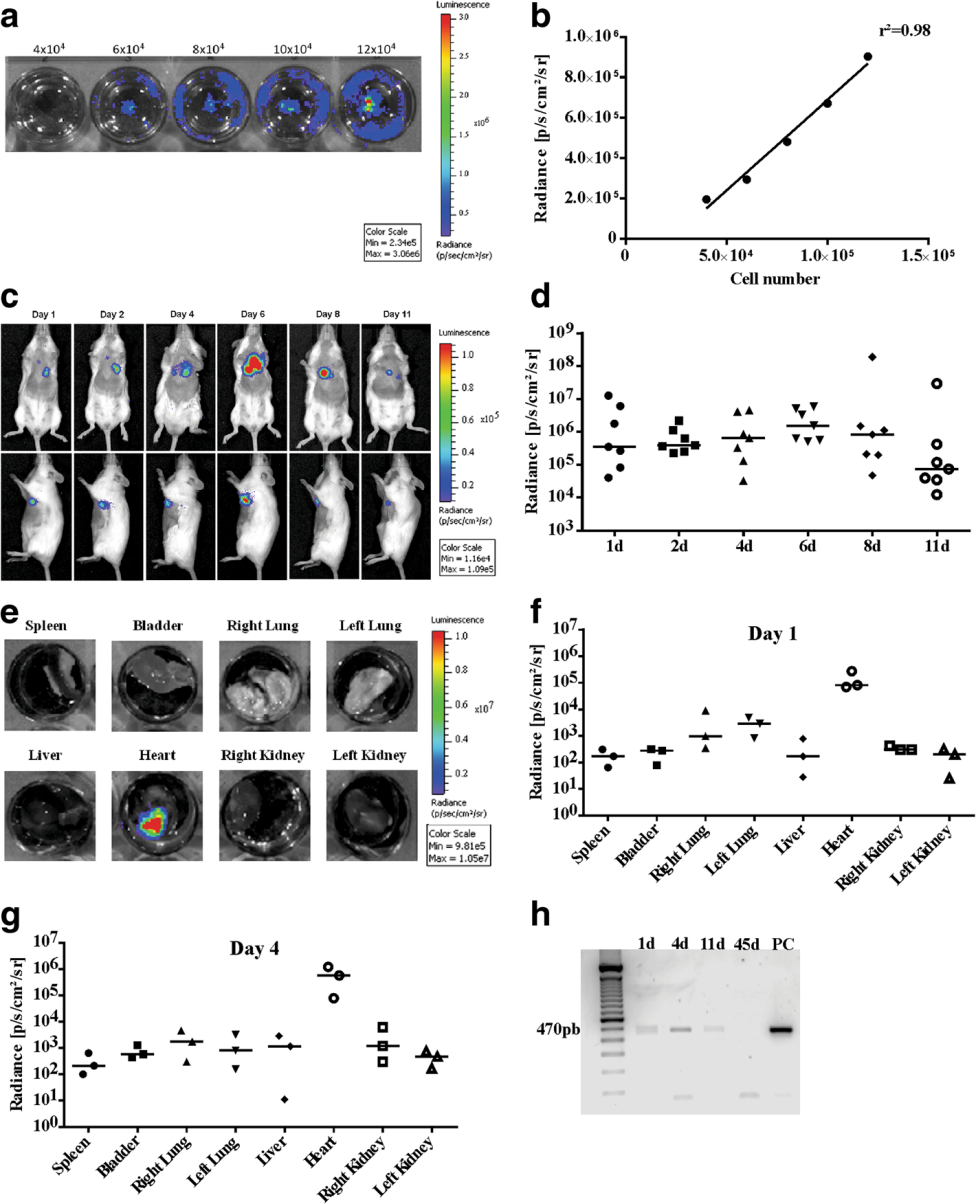
Cell tracking by bioluminescence. **a** Evaluation of luminescent signal intensity in CM-mESCs transduced with the luciferase 2 gene *in vitro*. Numbers shown above the wells indicate numbers of cells. **b** Signal increased with increasing cell number in a linear fashion (*r*^2^ = 0.98). **c** Representative images of CM-mESCs transduced with luciferase 2 after intramyocardial injection in DIC mice. Signal located in a region anatomically compatible with the heart in mice injected with CM-mESCs for up to 11 days after injection. **d** Quantification of luminescence in radiance units shows a decrease in the signal as time progresses. **e** *Ex-vivo* images and **f, g** quantification (*n* = 3 per day) of the luminescent signal demonstrating that cells remained in the heart at 1 day and 4 days after implantation, respectively. Signal lower in other organs (spleen, bladder, right lung, left lung, liver, right kidney and left kidney). **h** Presence of the luciferase 2 gene by PCR at 1, 4 and 11 days after cell injection. Luciferase 2 not observed at 45 days after injection. d days, PC positive control

CM-mESCs were transduced with a lentiviral vector containing the luciferase 2 and puromycin resistance genes under the control of an MSCV promoter. Using an IVIS Lumina Imaging System, we evaluated radiance *in vitro*. Imaging of a series containing a range of 4 × 104–12 × 10^4^ cells showed a strong linear correlation between cell number and radiance (*r*^2^ = 0.98; Fig. 5a, b), suggesting that this approach could reliably track and quantify cell distribution *in vivo*. The bioluminescent assays were carried out at 1, 2, 4, 6, 8, and 11 days after cell injection or until the luminescence signal disappeared. We observed that transplanted cells were detected in the thoracic region (Fig. 5c) of animals in the Dox + CM-mESC group (*n* = 7) from days 1 to 8, whereas at 11 days after cell transplantation the signal detected was approximately 80% weaker (Fig. 5d).

To confirm that cells were injected into the myocardium and remained in the heart, e*x-vivo* analysis was performed. Images of the different organs were obtained, and radiance was evaluated. Our results showed the presence of transplanted CM-mESCs E14TG2A-luc2 only in the hearts of mice (Fig. 5e) and at high intensity (8 × 10^4^ and 5.6 × 10^5^ radiance p/s/cm^2^/sr) at 1 day (*n* = 3) and 4 days (*n* = 3) after cell injection, respectively (Fig. 5f, g). Cells were also detected in the heart of one of the animals 11 days after cell therapy (see Additional file 1: Figure S1). Luminescence was undetectable in other organs. The luciferase 2 gene was detected via PCR at 1, 4 and 11 days after transplantation into the heart, but its expression was not observed at 45 days after cell therapy (Fig. 5h).

Immune rejection may explain the reduction in the number of cells from day 11 onward, since animals were treated with allogeneic cells in our experiments. To investigate this hypothesis, we performed a CM-mESC transplant in immunosuppressed CD1 mice, and cells were only detected until the 8th day after injection (see Additional file 2: Figure S2), indicating that immune rejection was not the cause of engraftment failure.

### Proteomic analysis of the secretome

The substantial cardiovascular recovery associated with the short-term retention of transplanted cardiomyocytes in the myocardium suggests secretion of paracrine mediators by the transplanted cells, rather than cellular engraftment. We therefore investigated the secreted proteins in the conditioned medium by LC-MS/MS-based proteomics. A total of 92 unique proteins were identified as described in Additional file 3: Table S1. The identified proteins were submitted to the Gene Ontology Biological Process, and seven proteins related to angiogenesis were observed, 10 proteins related to negative regulation of cell death and five proteins connected to the regulation of the reactive oxygen species metabolic process, among other cardiovascular recovery-related biological functions (see Additional file 4: Table S2).

### Quantification in myocardium collagen fiber

**Fig. 6:**
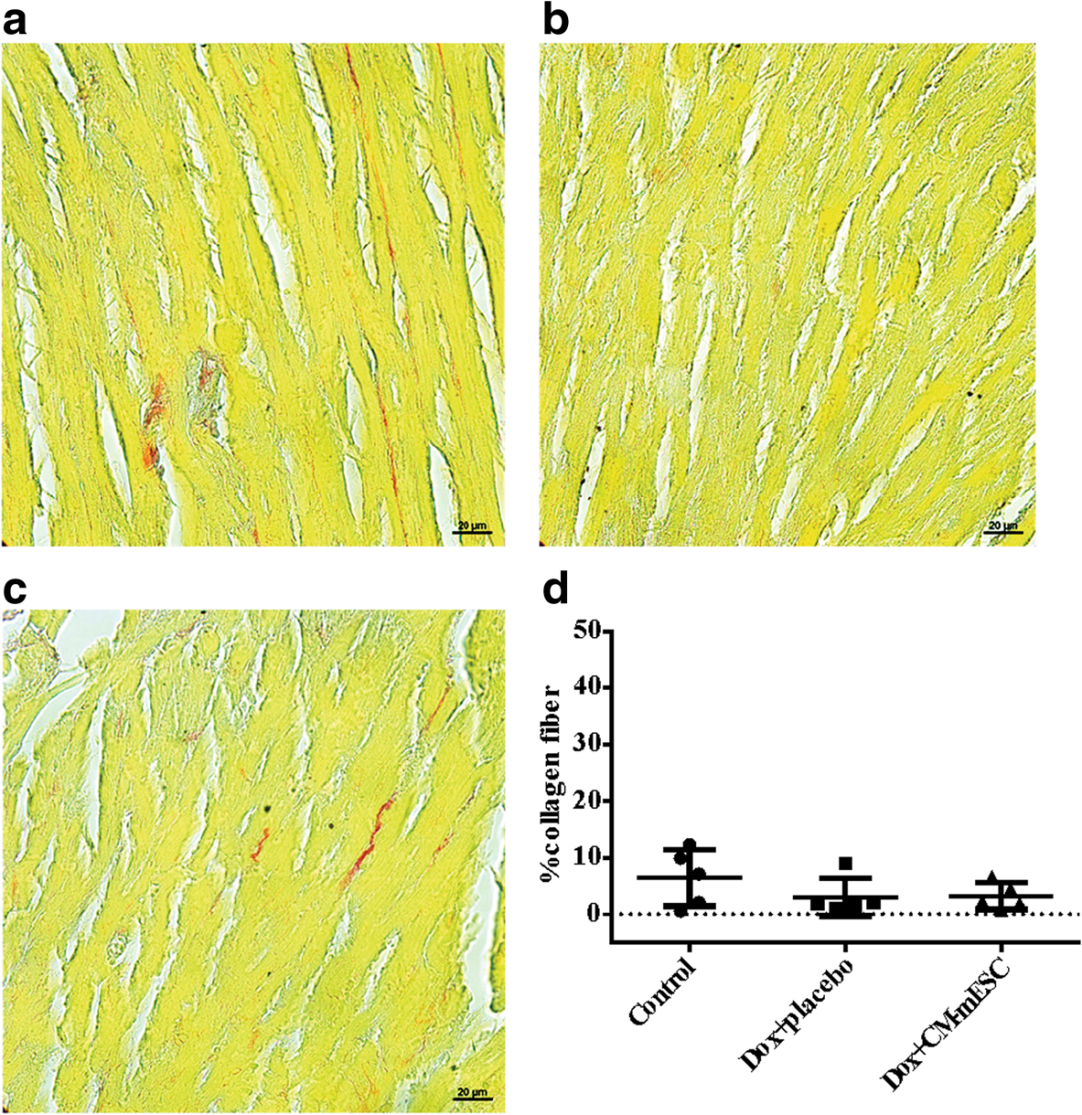
Histological analysis of the heart. **a**–**c** Representative images of histological sections stained with Sirius Red: **a** control group, **b** Dox + placebo group and **c** Dox + CM-mESC group after 45 days of cell therapy. **d** No changes in myocardial collagen fiber content. One-way ANOVA with a Bonferroni post test. Data shown as mean ± standard deviation; *n* = 5 for each group. Dox doxorubicin, CM-mESC cardiomyocytes derived from mouse embryonic stem cell

Histological analysis was performed on day 92 of the study design (corresponding to 45 days after cell or placebo injection). We did not observe any differences in the area occupied by collagen fibers in the hearts of animals among the control group (*n* = 5), the Dox + placebo group (*n* = 5) and the Dox + CM-mESC group (*n* = 5; Fig. 6a–d).

### Evaluation of apoptosis

**Fig. 7:**
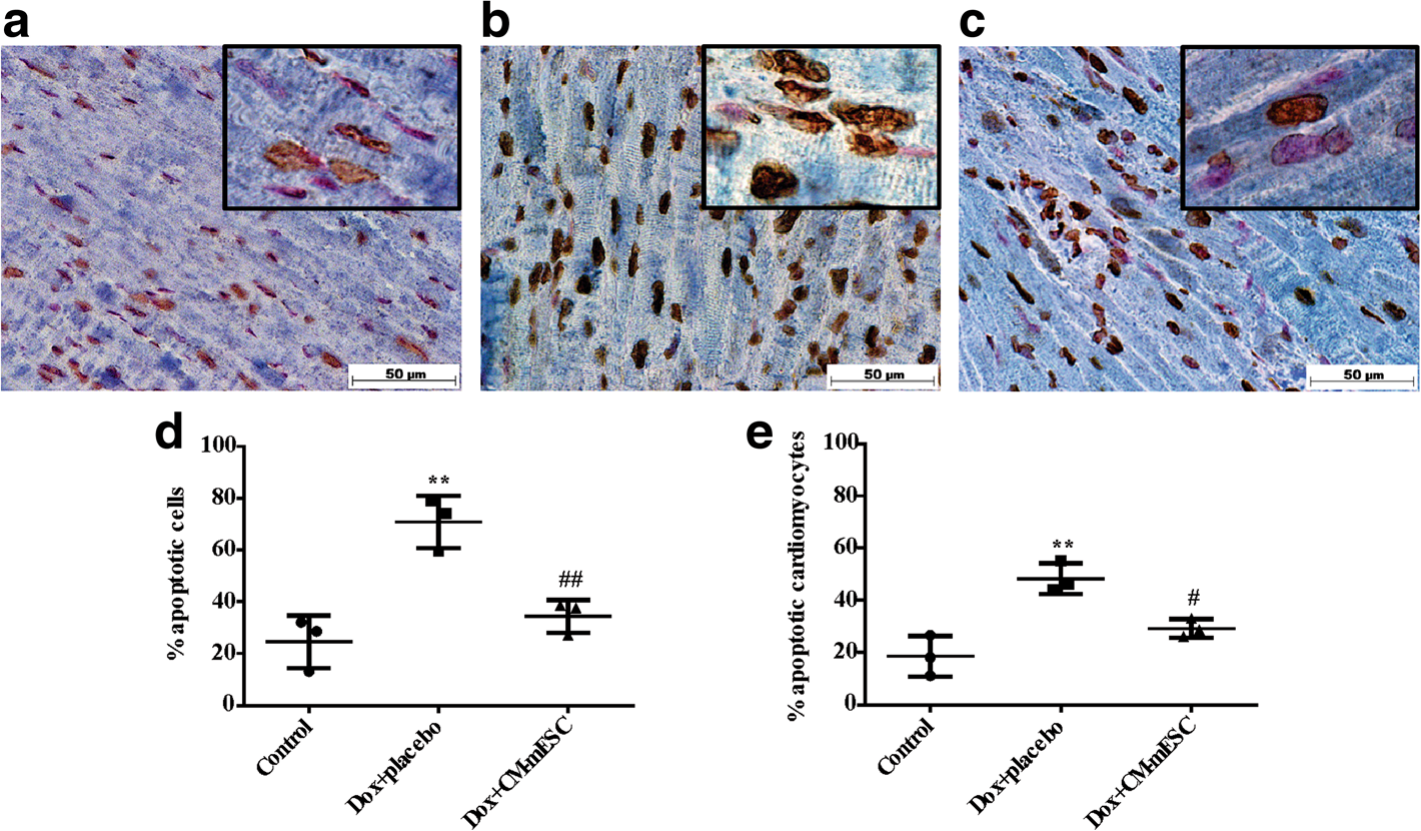
Apoptosis quantification in the heart. **a**–**c** Representative images of tissue sections stained with TUNEL (brown), cardiac troponin T (blue) and Safranin O (red) (nonapoptotic nuclei) at 45 days after cell therapy: **a** control group, **b** Dox + placebo group and **c** Dox + CM-mESC group. Quantification of **d** total apoptotic cells and **e** apoptotic cardiomyocytes. One-way ANOVA with a Bonferroni post test: **p < 0.01 compared to the control group; #*p* < 0.05, ##*p* < 0.01 compared to the Dox + placebo group. Data shown as mean ± standard deviation; *n* = 3 for each group. Dox doxorubicin, CM-mESC cardiomyocytes derived from mouse embryonic stem cell

To determine whether CM-mESC transplantation exerted an antiapoptotic effect, TUNEL staining was performed. We observed a significant reduction in the percentage of apoptotic cells and apoptotic cardiomyocytes in hearts of mice in the Dox + CM-mESC group (*n* = 3) when compared with the Dox + placebo group (*n* = 3). No significant difference was observed in this parameter between mice in the Dox + CM-mESC group and the control group (*n* = 3; Fig. 7a–e).

## Discussion

In this study, we showed that the intramyocardial transplantation of CM-mESCs restored cardiac function after DIC. Moreover, we established a mouse model of DIC using a new injection route for the drug.

Different groups that used the intraperitoneal administration route observed cardiac toxicity induced by Dox [19–22]. Additionally, one of the limitations of the mouse model in studies using Dox is the difficulty in obtaining repeated venous access through the animal’s tail due to local tissue injury caused by the drug [23]. Thus, we used the intracavitary route guided by echocardiography, and to our knowledge this is the only study so far to use this approach to develop an animal model of DIC. Moreover, in patients with cancer, Dox is administered intravenously and not intraperitoneally [5, 24]; therefore, our model more accurately reproduces the clinical setting.

A major concern of Dox therapy is related to the cumulative dose used, as it may cause heart toxicity. In this study, the total Dox dose tested (22.5 mg/kg BW) was selected based on the recommended therapeutic dose of 75 mg/m^2^ [25]. According to the formula described in the Guidance for Industry Document that converts doses administered to animals (mg/kg) to equivalent doses in humans (mg/m^2^), the 22.5 mg/kg BW dose of Dox corresponds to 66.6 mg/m^2^ in humans [26]. Furthermore, authors of previous studies have also obtained Dox-induced cardiotoxicity using doses equal to or similar to those used here [21, 24, 27]. Although DIC has been well described in preclinical and clinical studies, the underlying mechanism that induces cardiomyopathy is not fully understood. It is reported that DIC involves multifactorial mechanisms causing cellular death and fibrosis. These alterations trigger the remodeling process resulting in global cardiac dysfunction [1, 28, 29].

Despite its severe cardiotoxicity, Dox is still indicated for a variety of cancer treatments due to its important effect in curing neoplastic diseases [30]. In this scenario, several studies investigated transplantation of different cell types for the treatment of DIC in mice and rats [22, 31–35]. In contrast, our study used cardiomyocytes derived from embryonic stem cells, and this study is the only one that tracked transplanted cells *in vivo* using bioluminescence.

Prior to injection, we evaluated the efficiency of the differentiation protocol. The majority of mESCs differentiated into cardiomyocytes (mean 80%), but some of the cells differentiated into other cardiac cell types, such as fibroblasts, smooth muscle cells and, rarely, endothelial cells. After that, CM-mESCs were functionally evaluated in order to demonstrate their cardiac commitment. In consonance with the literature, the CM-mESCs presented electrophysiological properties similar to ventricular cardiomyocytes (APA and APD) [36, 37].

At 5 and 30 days after CM-mESC injection, we observed an improvement in the cardiac function of mice. Other researchers have also observed functional heart improvement in animals treated with undifferentiated induced pluripotent stem cells and embryonic stem cells [22, 34]. However, Nussbaum et al. [38] have shown that the presence of undifferentiated cells in a transplant could result in the formation of cardiac teratomas rather than new myocardium. Furthermore, other authors have shown that cell therapy with bone marrow and adipose-derived mesenchymal stem cells either failed or only partially improved the heart function of rats and rabbits with DIC [33, 35, 39, 40].

The electrocardiographic parameters showed complete recovery of the QT and QTc intervals on day 77 following transplantation of CM-mESCs. To date, only one study has evaluated the effect of cell therapy in cardiac electrical activity of animals (rabbits) with DIC and reported that the injected skeletal myoblasts exacerbate the delay and heterogeneity of ventricular electrical conduction, causing arrhythmias and fibrillation [39]. Different from these authors, we did not observe electrical conduction disturbances after cell therapy.

After CM-mESC transplantation, all animals survived until the end of the experimental protocol, showing that cell therapy was safe in this model. These results were similar to the findings by Yu *et al.* [35]*,* who observed that 100% of rats with DIC survived after cell therapy using bone marrow mesenchymal stem cells. However, these cells did not restore EF to normal values.

To explain the benefits of cellular therapy, two hypotheses are suggested: therapy may replace lost cardiomyocytes; and/or transplanted cells release factors capable of inducing cell proliferation, angiogenesis and promoting cardioprotective effects, the so-called “paracrine effect” [12, 41, 42].

To verify whether CM-mESCs engraft on heart tissue in the hearts of animals that present with DIC, a bioluminescence assay was performed to track the cells. Our results showed that cells could be found until the 11th day after injection. Over this period, we observed an 80% drop in luminescence intensity (Fig. 5c). In agreement with our data, Wu’s group observed the presence of cardiomyocytes derived from human embryonic stem cells at up to 56 days after transplantation in the hearts of infarcted mice; however, the bioluminescence signal was very weak (90% decay) from the 10th day onward [42].

Using adult multipotent cells, Cao *et al.* [43] have shown that bone marrow mesenchymal cells remained for up to 6 days in the heart region when cells were transplanted in infarcted rats. In addition, Passipieri *et al.* [12] detected placental-derived mesenchymal stem cells in the thoracic region of infarcted rats for 3 days after transplantation, despite using immunosuppressive therapy.

We also used immunosuppression and could not prolong cell detection by in-vivo bioluminescence. The loss of the bioluminescence signal may be explained either by an absence of cells or by a low number of remaining cells, which results in the signal falling below the lower limit of detection of the equipment [44]. We do not believe that this can be explained by signal fading, since the cells were transduced with a stable lentiviral vector that is expressed constitutively. Further evidence supporting the absence of engraftment of transplanted cells was given by the lack of luciferase gene detection by PCR, which is a very sensitive assay.

Our data suggest that the benefits of improved heart function were induced by paracrine effects, since the cells could not be detected in the hearts beyond 11 days after injection. The proteomic profile of the CM-mESC-conditioned medium reinforces a paracrine effect because antiapoptotic, angiogenic and antioxidant proteins were identified in TCM.

Different authors have shown that Dox induces fibrosis in the hearts of mice, rats and rabbits [22, 31, 45, 46]. In addition, some authors have shown that cell therapy decreases the amount of fibrosis in the heart tissue of these animals [22, 45]. In contrast to these data, in our study we did not observe an increase in the amount of fibrosis after Dox administration. In line with our results, Desai *et al*. [24] also showed cardiac dysfunction after Dox administration in B6C3F1 mice with a low degree of injury to the heart. In addition, Farhad *et al.* [47] demonstrated that fibrosis is not an early event in the mouse model of DIC*.* More recent studies have shown a decrease in capillary density and reduced vascular tree branching in the hearts of mice after Dox administration [48, 49], suggesting that the rarefaction of microcirculation may also explain the development of Dox-induced heart failure. According to Sun *et al*. [49], microvascular deficit occurs before fibrosis formation and is considered a crucial factor in the evolution of a functionally decompensated cardiac status. We speculate that significant fibrosis might have developed at longer time intervals after Dox injection.

Cardiomyocyte death due to apoptosis is a significant contributor to the development and progression of DIC [1, 22] and is considered an early molecular event of cardiac tissue damage [50]. Hence, we measured apoptosis in heart tissue using a TUNEL assay. We demonstrated that hearts transplanted with CM-mESCs have a significantly reduced percentage of apoptotic cardiomyocytes in the heart, which is a mechanism that likely contributes to the observed improvement of cardiac function. Importantly, proteomics demonstrated that CM-mESCs secrete proteins that negatively regulate cell death, further supporting a paracrine role for these cells in the reduction of Dox-induced apoptosis.

## Conclusions

We have developed a new model for DIC utilizing an intracavitary delivery of Dox and have shown that CM-mESCs injected into the ventricular walls of DIC mice restored cardiac function, most likely through paracrine mechanisms.

## Additional files

Additional file 1: Figure S1. Showing *ex-vivo* images of the luminescent signal at 11 days after cell therapy. (**a**) Cells remained in the heart. (**b**) Signal lower in other organs (spleen, bladder, right lung, left lung, liver, right kidney and left kidney). (TIFF 5947 kb)

Additional file 2: Figure S2. Showing cell tracking by bioluminescence in immunosuppressed CD1 mice. **(a)** Representative images of CM-mESCs transduced with luciferase 2 after intramyocardial injection in immunosuppressed CD1 mice. Signal located in a region anatomically compatible to the heart in mice injected with CM-mESCs up to 8 days after injection. **(b)** Quantification of luminescence in radiance units shows a decrease in signal as time progresses. (TIFF 6143 kb)

Additional file 3: Table S1. Presenting a list of 92 unique proteins identified by LC-MS/MS. (DOCX 14 kb)

Additional file 4: Table S2. Presenting a list of Gene Ontology Biological Processes of interest. (DOCX 12 kb)

## Abbreviations

ANOVA: Analysis of variance
APD: Action potential duration
BSA: Bovine serum albumin
BW: Body weight
CID: Collision-induced dissociation
CM-mESCs: Cardiomyocytes derived from mouse embryonic stem cells
DAB: 3,3′-Diaminobenzidine
DDA: Data-dependent automatic
DIC: Dox-induced cardiomyopathy
DMEM: Dulbecco’s Modified Eagle’s Medium
DMSO: Dimethyl sulfoxide
Dox: Doxorubicin
DTT: Dithiothreitol
EB: Embryoid body
ECG: Electrocardiogram
ECHO: Echocardiogram
EDV: End-diastolic volume
EF: Ejection fraction
ESV: End-systolic volume
FBS: Fetal bovine serum
FDR: False discovery rate
GMEM: Glasgow Minimum Essential Medium
HR: Heart rate
IAM: Iodoacetamide
IC: Intracavitary route
LC-MS/MS: Liquid chromatography tandem mass spectrometry
LIF: Leukemia inhibitory factor
Luc2: Luciferase 2
mESC: Mouse embryonic stem cell
OCT3/4: Octamer-binding transcription factor 3/4
PCR: Polymerase chain reaction
PSM: Peptide sequence match
QTc: Corrected QT
SSEA-1: Stage-specific embryonic antigen-1
SV: Stroke volume
TCM: Total conditioning medium
TUNEL: Deoxynucleotidyl transferase-mediated 2′-deoxyuridine 5′-triphosphate nick end labeling
