# Supplementary figures and images for: Embryonic stem cell-derived cardiomyocytes for the treatment of doxorubicin-induced cardiomyopathy
Source: Stem Cell Res Ther. 2018 Feb 5;9:30. doi: 10.1186/s13287-018-0788-2 (PMC5799903; doi:10.1186/s13287-018-0788-2)

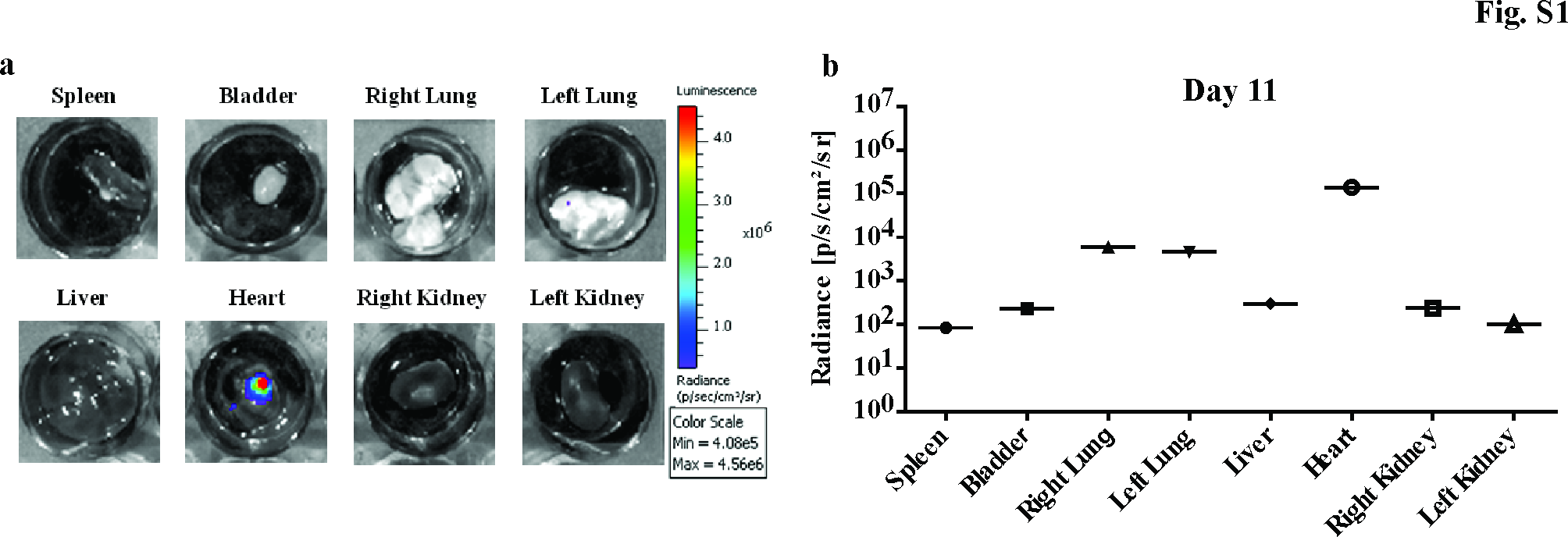

Supplement: Supplementary file 1 — Showing ex-vivo images of the luminescent signal at 11 days after cell therapy. (a) Cells remained in the heart. (b) Signal lower in other organs (spleen, bladder, right lung, left lung, liver, right kidney and left kidney). (TIFF 5947 kb) [file 13287_2018_788_MOESM1_ESM.tif]

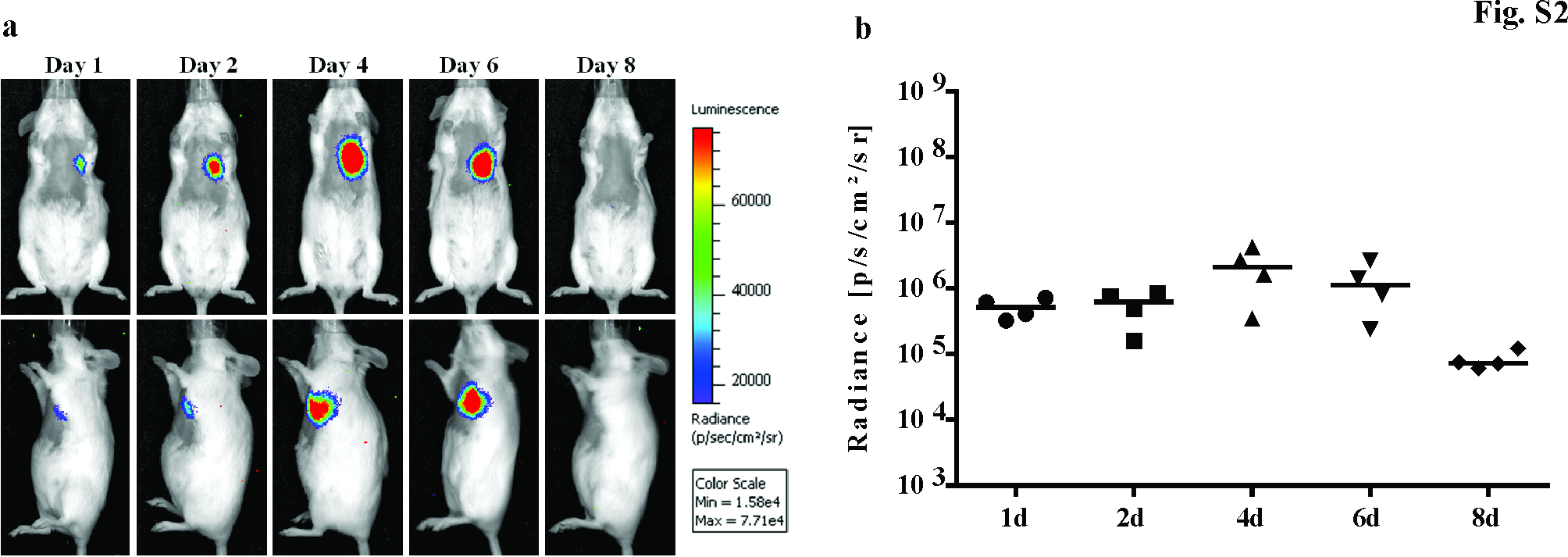

Supplement: Supplementary file 2 — Showing cell tracking by bioluminescence in immunosuppressed CD1 mice. (a) Representative images of CM-mESCs transduced with luciferase 2 after intramyocardial injection in immunosuppressed CD1 mice. Signal located in a region anatomically compatible to the heart in mice injected with CM-mESCs up to 8 days after injection. (b) Quantification of luminescence in radiance units shows a decrease in signal as time progresses. (TIFF 6143 kb) [file 13287_2018_788_MOESM2_ESM.tif]
