# Supplementary material for: Embryonic stem cell-derived cardiomyocytes for the treatment of doxorubicin-induced cardiomyopathy
Source: Stem Cell Res Ther. 2018 Feb 5;9:30. doi: 10.1186/s13287-018-0788-2 (PMC5799903; doi:10.1186/s13287-018-0788-2)
Supplement: Supplementary file 3 — Presenting a list of 92 unique proteins identified by LC-MS/MS. (DOCX 14 kb) [file 13287_2018_788_MOESM3_ESM.docx]

**Table S1. List of 92 unique proteins identified by LC-MS/MS.**

| FBLN2 | Fibulin-2 |
| --- | --- |
| CO4A2 | Collagen alpha-2(IV) chain |
| TTHY | Transthyretin |
| PTK7 | Inactive tyrosine-protein kinase 7 |
| BGH3 | Transforming growth factor-beta-induced protein ig-h3 |
| LEG1 | Galectin-1 |
| EF2 | Elongation factor 2 |
| GDIR1 | Rho GDP-dissociation inhibitor 1 |
| CATL1 | Cathepsin L1 |
| CATB | Cathepsin B |
| NID2 | Nidogen-2 |
| FSCN1 | Fascin |
| AEBP1 | Adipocyte enhancer-binding protein 1 |
| G6PI | Glucose-6-phosphate isomerase |
| TKT | Transketolase |
| PDIA3 | Protein disulfide-isomerase A3 |
| POSTN | Periostin |
| CO3 | Complement C3 |
| CO6A1 | Collagen alpha-1(VI) chain |
| NUCL | Nucleolin |
| IBP3 | Insulin-like growth factor-binding protein 3 |
| FBLN1 | Fibulin-1 |
| EF1A1 | Elongation factor 1-alpha 1 |
| FETA | Alpha-fetoprotein |
| EF1G | Elongation factor 1-gamma |
| NID1 | Nidogen-1 |
| FSTL1 | Follistatin-related protein 1 |
| PSA2 | Proteasome subunit alpha type-2 |
| FLNB | Filamin-B |
| CO1A2 | Collagen alpha-2(I) chain |
| GDIB | Rab GDP-dissociation inhibitor beta |
| SODC | Superoxide dismutase [Cu-Zn] |
| CAD11 | Cadherin-11 |
| PEPA5 | Pepsin A-5 |
| PLAK | Junction plakoglobin |
| HSP7C | Heat shock cognate 71 kDa protein |
| HS71B | Heat shock 70 kDa protein 1B |
| ENOA | Alpha-enolase |
| FLNA | Filamin-A |
| A0A1L1SSH9 | SPARC |
| CLUS | Clusterin |
| CO3A1 | Collagen alpha-1(III) chain |
| FINC | Fibronectin |
| LDHA | L-lactate dehydrogenase A chain |
| PRDX2 | Peroxiredoxin-2 |
| H2B1K | Histone H2B type 1-K |
| ACTN1 | Alpha-actinin-1 |
| TENA | Tenascin |
| ALDOA | Fructose-bisphosphate aldolase A |
| ACTB | Actin, cytoplasmic 1 |
| ACTA | Actin, aortic smooth muscle |
| ACTBL | Beta-actin-like protein 2 |
| E9PWQ3 | Collagen, type VI, alpha 3 |
| PGAM1 | Phosphoglycerate mutase 1 |
| KPYM | Pyruvate kinase PKM |
| PCOC1 | Procollagen C-endopeptidase enhancer 1 |
| PXDN | Peroxidasin homolog |
| LAMA1 | Laminin subunit alpha-1 |
| F8VQ40 | Laminin subunit alpha-1 |
| TBA1A | Tubulin alpha-1A chain |
| GELS | Gelsolin |
| CATD | Cathepsin D |
| CSPG2 | Versican core protein |
| Q8BS97 | Putative uncharacterized protein |
| NDKA | Nucleoside diphosphate kinase A |
| G3X977 | Inter-alpha trypsin inhibitor, heavy chain 2 |
| MMP2 | 72 kDa type IV collagenase |
| Q3U2G2 | Heat shock 70 kDa protein 4 |
| TCTP | Translationally controlled tumor protein |
| PPIA | Peptidyl-prolyl cis-trans isomerase A |
| Q80YQ1 | Thrombospondin 1 |
| HS90B | Heat shock protein HSP 90-beta |
| HS90A | Heat shock protein HSP 90-alpha |
| LAMC1 | Laminin subunit gamma-1 |
| F8VQJ3 | Laminin subunit gamma-1 |
| DESP | Desmoplakin |
| CADH2 | Cadherin-2 |
| CO5A1 | Collagen alpha-1(V) chain |
| CO4A1 | Collagen alpha-1(IV) chain |
| TPIS | Triosephosphate isomerase |
| CO1A1 | Collagen alpha-1(I) chain |
| CO5A2 | Collagen alpha-2(V) chain |
| NPM | Nucleophosmin |
| LAMB1 | Laminin subunit beta-1 |
| PGK1 | Phosphoglycerate kinase 1 |
| A0A1W2P768 | Histone H3.2 |
| B1B0C7 | Basement membrane-specific heparan sulfate proteoglycan core protein |
| PGBM | Basement membrane-specific heparan sulfate proteoglycan core protein |
| E9Q035 | Uncharacterized protein |
| E9QPX1 | Collagen alpha-1(XVIII) chain |
| E9Q718 | Procollagen-lysine,2-oxoglutarate 5-dioxygenase 2 |
