# Supplementary material for: Embryonic stem cell-derived cardiomyocytes for the treatment of doxorubicin-induced cardiomyopathy
Source: Stem Cell Res Ther. 2018 Feb 5;9:30. doi: 10.1186/s13287-018-0788-2 (PMC5799903; doi:10.1186/s13287-018-0788-2)
Supplement: Supplementary file 4 — Presenting a list of Gene Ontology Biological Processes of interest. (DOCX 12 kb) [file 13287_2018_788_MOESM4_ESM.docx]

**Table S2. List of Gene Ontology Biological Processes of interest.**

| #pathway ID | pathway description | observed gene count | false discovery rate | matching proteins in your network (IDs) |
| --- | --- | --- | --- | --- |
| GO.0001944 | vasculature development | 14 | 6.26×10^-7^ | Acta2, Cdh2, Col1a1, Col1a2, Col3a1,  Col4a1, Col4a2, Col5a1, Fn1, Gpi1, Lama1,  Mmp2, Ncl, Tgfbi |
| GO.0072358 | cardiovascular system development | 16 | 0.0000036 | Acta2, Cdh2, Col1a1, Col1a2, Col3a1,  Col4a1, Col4a2, Col5a1, Dsp, Fn1, Gpi1,  Lama1, Mmp2,  Ncl, Tgfbi, Vcan |
| GO.0072359 | circulatory system development | 16 | 0.0000036 | Acta2, Cdh2, Col1a1, Col1a2, Col3a1,  Col4a1, Col4a2, Col5a1, Dsp, Fn1, Gpi1,  Lama1, Mmp2,  Ncl, Tgfbi, Vcan |
| GO.0048514 | blood vessel morphogenesis | 10 | 0.000144 | Cdh2, Col3a1, Col4a1, Col4a2, Fn1, Gpi1,  Lama1, Mmp2, Ncl, Tgfbi |
| GO.0001525 | angiogenesis | 7 | 0.0074 | Col4a1, Col4a2, Fn1, Gpi1, Mmp2, Ncl, Tgfbi |
| GO.2000377 | regulation of reactive oxygen species metabolic process | 5 | 0.0105 | Clu, Hsp90aa1, Hsp90ab1, Prdx2, Sod1 |
| GO.0051764 | actin crosslink formation | 2 | 0.026 | Actn1, Flna |
| GO.0060548 | negative regulation of cell death | 10 | 0.0303 | Clu, Ctsb, Flna, Hsp90ab1, Ncl, Nme1, Npm1,  Prdx2, Sod1, Tpt1 |
| GO.0080134 | regulation of response to stress | 11 | 0.0489 | C3, Clu, Gsn, Hsp90ab1, Mmp2, Nme1,  Npm1, Prdx2, Psma1, Sod1, Tpt1 |
